# Outcomes of Woodward’s Procedure for Sprengel’s Shoulder Using Neurophysiological Monitoring of the Brachial Plexus Without Clavicular Osteotomy: A Retrospective Study

**DOI:** 10.7759/cureus.19797

**Published:** 2021-11-21

**Authors:** Ozair Bin Majid, Saleh z Alzaid, Zayed Al-Zayed, Shahd Almonaie, Alanoud A Albekairi, Maqsood Ahmed

**Affiliations:** 1 Orthopaedic Surgery, King Faisal Specialist Hospital and Research Centre, Riyadh, SAU; 2 Orthopaedic Surgery, King Fahad Medical City, Riyadh, SAU; 3 Orthopaedic Surgery, Alfaisal University College of Medicine, Riyadh, SAU; 4 Orthopaedics, Alfaisal University College of Medicine, Riyadh, SAU; 5 Neurophysiology, King Faisal Specialist Hospital and Research Centre, Riyadh, SAU

**Keywords:** cavendish grade, scapula, neurophysiological monitoring, brachial plexus, woodward's procedure, sprengel deformity

## Abstract

Introduction

For Sprengel deformity, a variety of operations are available, with Woodward's procedure being a favorable option with good outcomes. This study aims to assess the outcomes of Woodward's procedure with brachial plexus monitoring to prevent the possible complications of nerve injury and consequent deficits.

Methods

In our study, we included 18 patients with Sprengel deformity treated with Woodward's procedure using intraoperative neuromonitoring for the brachial plexus from 2013 to 2019 at our institute. For each patient, we collected information about age, gender, follow-up duration, affected shoulder side, and presence of an omovertebral bar. Also, preoperative and postoperative degrees of shoulder abduction, Cavendish grade of cosmetic appearance, Rigaults grade, and difference in scapular elevation along with postoperative complications were all measured to evaluate the outcomes.

Results

The mean duration of follow-up was 12 months. The average preoperative Cavendish grade was 3.1, which decreased to 1.3 on the final follow-up. The average preoperative Rigault grade was 2.5, which has decreased to an average of 1.8. The average increase in the degree of shoulder abduction postoperatively was 48.3°. The average preoperative difference in scapular height (mm) was 26.9, which decreased to an average of 12.2. Furthermore, the final outcome was not impacted by the absence or the existence of the omovertebral bar.

Conclusion

Woodward's procedure using intraoperative neuromonitoring without clavicle osteotomy for Sprengel's deformity successfully corrects the deformity and decreases the risk of iatrogenic brachial plexus injury.

## Introduction

Back in 1891, Sprengel was the first to describe the abnormal elevation of the scapula; thus, the condition was termed with reference to his name [1]. Sprengel deformity is a developmental abnormality of the scapula, leading to its elevation from its physiological bed. Further, it is the most common shoulder girdle developmental anomaly. Sprengel deformity severity differs on a scale that ranges from very mild, mild, moderate, and severe based on the Cavendish classification with surgical management being indicated only for severe cases. Woodward’s procedure is a preferred approach technique and has a decreased potential risk of iatrogenic brachial plexus injury and less bleeding. Surgical correction places a potentiality for complications to occur, including brachial plexus injury, winging of the scapula, hypertrophic scar, and regrowing of the scapular superior pole. The complication that is the highest in severity is the brachial plexus injury, leading to paresis of the upper extremity. Complications that could occur in surgical management range from 12.5% to 58.7% [1-8]. Intraoperative neurophysiological monitoring plays an important role during the surgical correction of Sprengel deformity to monitor the brachial plexus function. Multiple neurophysiological modalities, such as somatosensory evoked potential (SSEP), motor evoked potential (MEP), free-running electromyogram (FEMG), and electroencephalogram (EEG), are used to avoid the adverse outcomes of the procedure. Although SSEP monitoring provides real-time data on neuropraxia on the nerves of the brachial plexus, MEP is more sensitive for identifying any changes, and the feedback is instantaneous [2-4].

## Materials and methods

In our study, we included 18 with a congenital elevation of the scapula surgically treated for Sprengel deformity by Woodward’s procedure using intraoperative neurophysiological monitoring from 2013 to 2019 at King Faisal Specialist Hospital, Riyadh, Saudi Arabia. The study data were collected from the hospital's electronic database records.

Consent was waived for all participants in this study. This study was approved by the Institutional Review Board of King Faisal Hospital and Research Center, Riyadh, Saudi Arabia.

Age, gender, follow-up duration, affected shoulder side, and the presence of the omovertebral bar were all noted. In addition, preoperative and postoperative degrees of shoulder abduction, Cavendish grade of cosmetic appearance, radiographic imaging, and Rigaults grade thereafter, the difference in scapular elevation in mm, along with postoperative complications, were all measured to evaluate the outcomes. There were seven males and 11 females. The average age at when the procedure was conducted is four years and nine months. However, we have included an adult patient aged 24 years to get more insight into this deformity when it enters the phase of adulthood and its management principles. Thereby, the average age when including the adult patient is five years and 10 months. The patients were evaluated and managed in the orthopedic surgery department of our institution and all the surgeries were conducted by Dr. Zayed Saleh Alzayed. The minimum follow-up duration was seven months and the maximum was 48 months with an average follow-up period of 12 months. Any major or minor complications of the procedure were taken note of.

Preoperative evaluation

We evaluated the patients visiting our hospital using the Cavendish grading system and measured the degree of shoulder abduction. After the clinical evaluation, plain radiographs are ordered to assess the relation of the scapula’s level to the vertebrae along with the contralateral scapula. We also used a CT scan with three-dimensional reconstruction in all our cases to assess the presence of the omovertebral bar and associated congenital anomalies of the vertebrae.

Surgical technique

**Figure 1:**
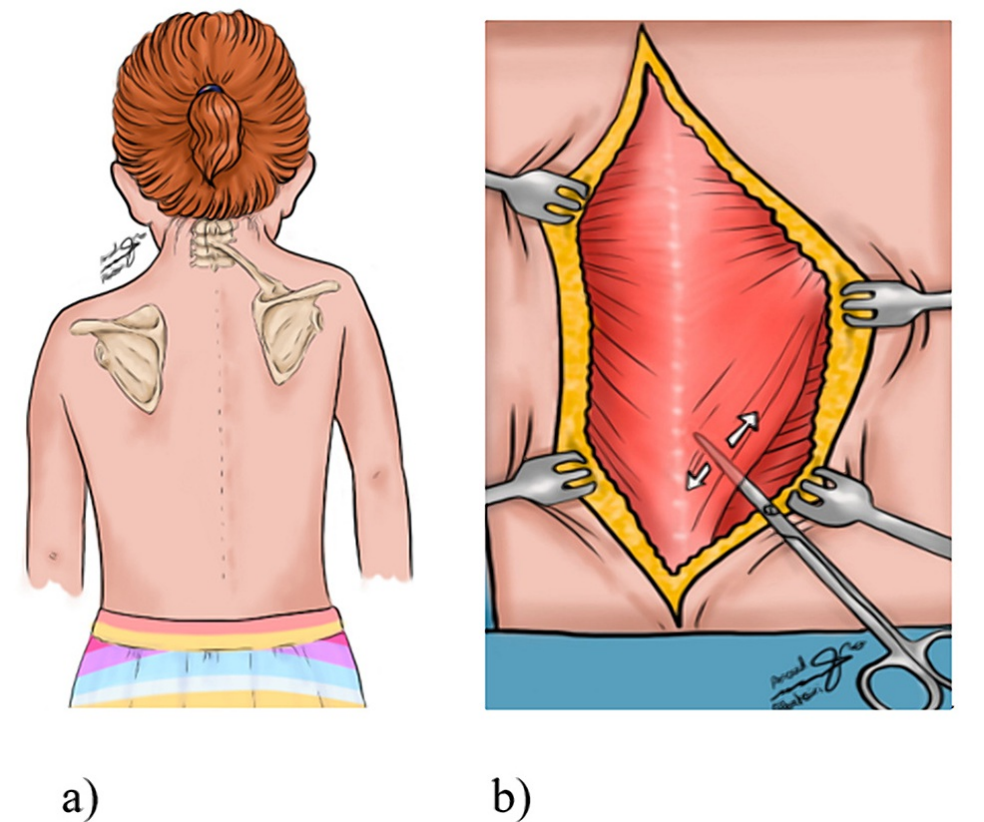
Sprengel deformity a) Sketch showing Sprengel shoulder and the presence of the omovertebral bar in the left side. b) Sketch showing Woodward’s procedure. The lower portion of the trapezius muscle is separated from the underlying Latissimus dorsi muscle using Metzenbaum scissors.

**Figure 2:**
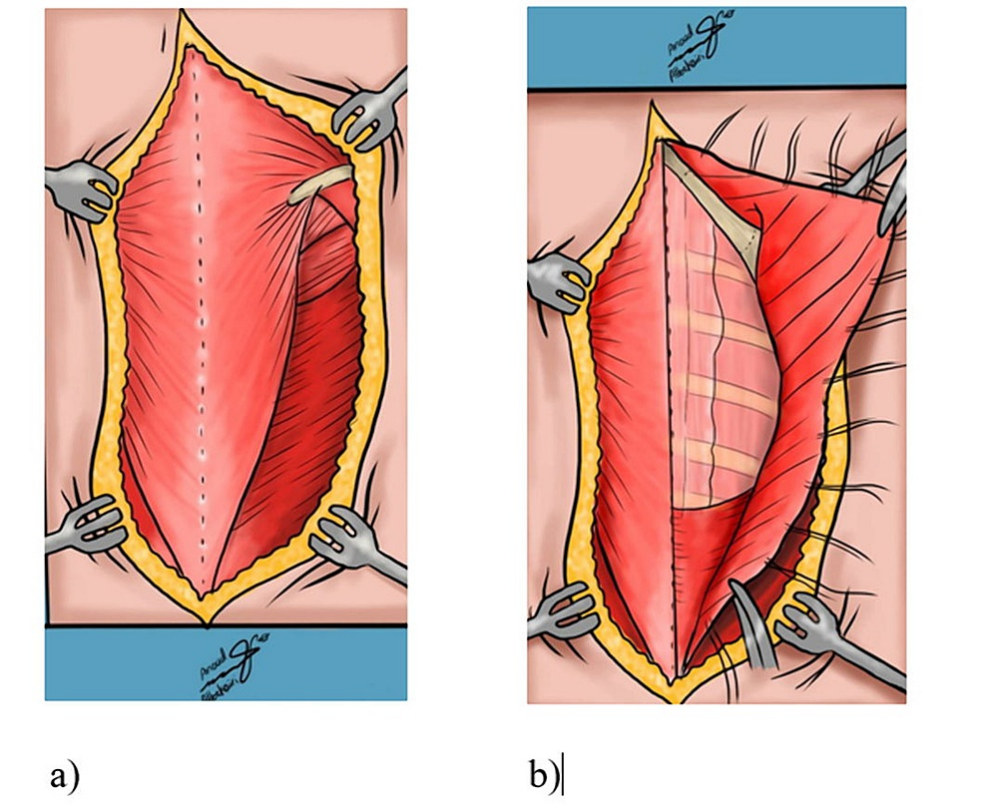
Woodward's procedure a) The Trapezius muscle is shown here where it is attached to the spinous processes of C1-C2 to T9, as well as the spine of the scapula. b)The omovertebral bar is shown while the entire muscle sheet is retracted laterally, with lines that show the surgical excision sites using a bone cutter.

**Figure 3:**
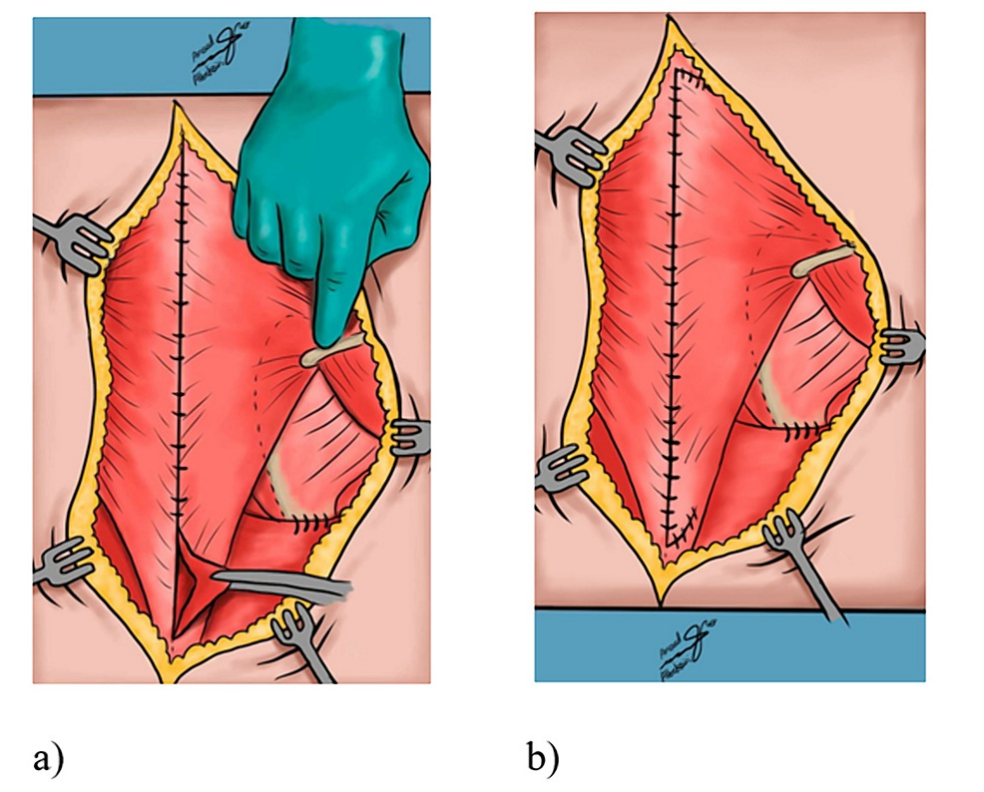
Sketches of Woodward's procedure a) The rhomboids and the aponeurosis of the Trapezius muscle are attached again to the spinous processes on a lower level and sutured. b) The latissimus dorsi muscle is also reattached at its origin with the scapula depressed.

Woodward’s technique done on our reviewed patients was performed with the patient in a prone position. Draping was done in a manner so that we could easily manipulate the involved side shoulder girdle. After general anesthesia, intraoperative neurophysiological monitoring was employed to monitor the brachial plexus on the involved side and baseline measurement was obtained. The procedure involved a linear longitudinal midline incision from the cervical region to the lower region of the thoracic vertebrae. Both the trapezius muscle and the rhomboids are dissected subperiosteally and are further retracted from the spinous processes they originate from. The scapula was displaced inferiorly after disconnecting possible omovertebral connection through extra-periosteal dissection. After replacing the scapula caudally by pulling it downwards and rotating it so that the spinous processes of the two scapulae are at the same level, the muscles are reattached to the vertebrae with the new scapula in its physiological position (Figures 1-3) [6].

Intraoperative neuromonitoring

Intraoperative neuromonitoring was done using the Nicolet Endeavor CR IOM machine. In SSEP monitoring, median or ulnar nerve stimulation on both upper limbs was done at the wrist using a square wave of 200µs with the rate of 4.7 per second and 15-25ma intensity. Cortical responses from contralateral primary sensory cortices are measured with the derivation of CP3-CPZ and CP4-CPZ, and peripheral response is obtained from the cubital fossa medial to the biceps tendon with a high-frequency filter of 1500 Hz and low-frequency filter of 30 Hz and a sweep time of 30 ms windows. The criteria to identify significant degradation of the SEPs is amplitude reduction of 50% from the baseline and/or latency prolongation of 10% from the baseline. MEP is more sensitive, as no averaging is required due to very high signal-to-noise ratios (SNRs) and the feedback is instantaneous. Transcranial electrodes are placed for stimulation, and recording is done from muscles such bilateral deltoid, biceps, brachioradialis, and thenar muscles with a train of stimuli of five impulses and inter-pulse interval of 1 ms. Free-running electromyography (EMG) was used to give instantaneous feedback during the procedure. Any mechanical irritation of the nerve innervating the deltoids, biceps, and brachioradialis could be detected by this modality. Finally, EEG shows cortical activity and is used to detect gross changes in cortical function. However, during anesthesia, the depth of anesthesia affects the EEG activity [2-4].

Statistical analysis

All statistical analyses were carried out using the SPSS V23 software package (IBM Corp., Armonk, NY). The normality of all variables was assessed using the Kolmogorov-Smirnov test. Continuous variables were presented as mean ± standard deviation and median (quartile 1, quartile 3) for normal and non-normal variables, respectively. Categorical variables were presented as frequency (%). The range for continuous variables was presented as a minimum-maximum data point. Change and % change at follow-up post-Woodward's procedure was calculated using the preop. and follow-up data for improvement metrics, and the statistical significance was calculated using a paired sample t-test. Bivariate correlation between age at surgery and improvement metrics from the Woodward procedure was calculated and presented as Spearman’s correlation coefficient (r). The difference in the improvement post-Woodward's procedure in the two groups (divided by an Omvertal bar) was calculated using an independent student t-test and Mann-Whitney U-test for continuous normal and non-normal variables, respectively. In all the statistical tests, p<0.05 was considered significant. The figures were plotted in Microsoft Excel (Microsoft Corporation, Redmond, NY). 

## Results

**Figure 4:**
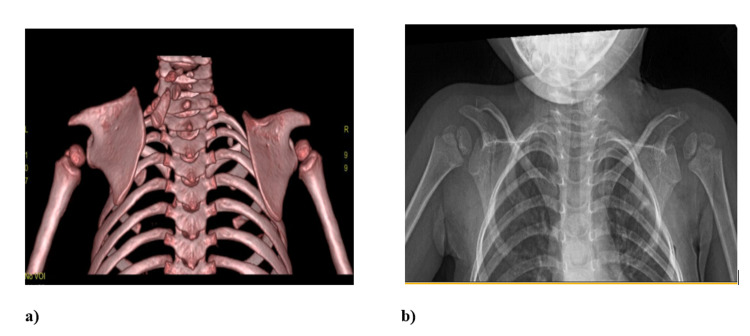
a) CT scan of a three-year-old patient with Sprengel's shoulder and omovertebral bone. b) Plain radiograph of the same three-year-old patient showing correction of Sprengel's deformity at follow-up post-Woodward’s procedure

Eleven females (61.1%) and five males (38.9%) were operated on at a mean age of four years (range: 2-24 years). The left and right shoulders that were operated on were 14 (77.8%) and four (22.2%), respectively. The mean follow-up duration was 12 months (range: 7-48 months). Moreover, omovertebral bar presence was positive in 14 (77.8%) of the patients while only four (22.2%) of the patients were negative for it (Table 1). Figure 4 shows the correction of Sprengel deformity with an omovertebral bar in a three-year-old patient.

**Table 1:** Difference in improvement post-Woodward's procedure in two groups divided by the omovertebral bar Note: Data are shown as mean ± S.D. and range for normal continuous variables ($), and median (25th percentile, 75th percentile) and range for non-normal continuous variables (#).

Improvement metrics	Positive (N=14)	Negative (N=4)	p
Age at surgery (years) #	4.0 (3, 6)	6.5 (4, 16.5)	0.19
Cavendish grade improvement $	1.79 ± 0.6	1.75 ± 1	0.95
Renault grade improvement $	0.71 ± 0.6	0.50 ± 0.6	0.55
Improvement in shoulder abduction $	47.86 ± 23.6	50.0 ± 10.8	0.81
Improvement in the difference of scapular height $	14.43 ± 4.8	15.75 ± 5.9	0.65

**Figure 5:**
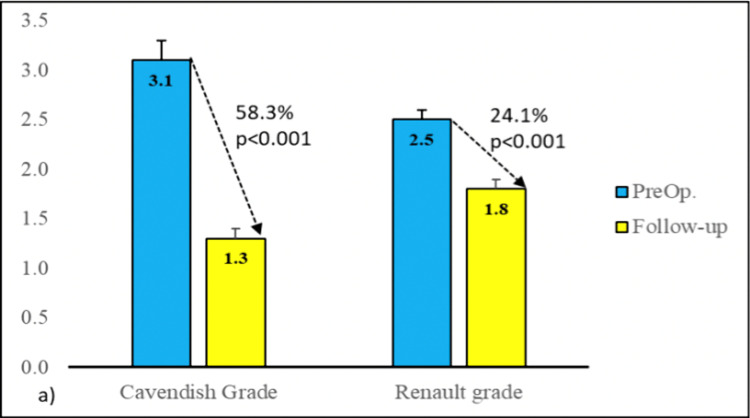
Change in the Cavendish grade and Rigaults grade from the preoperative period and post-surgical correction

**Figure 6:**
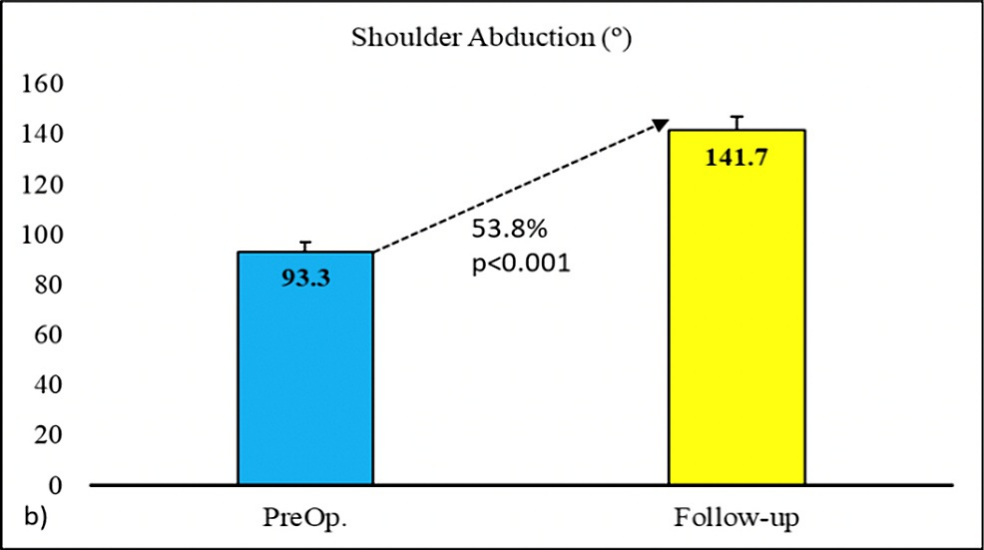
Change in shoulder abduction from preoperative stage to postoperative stage

**Figure 7:**
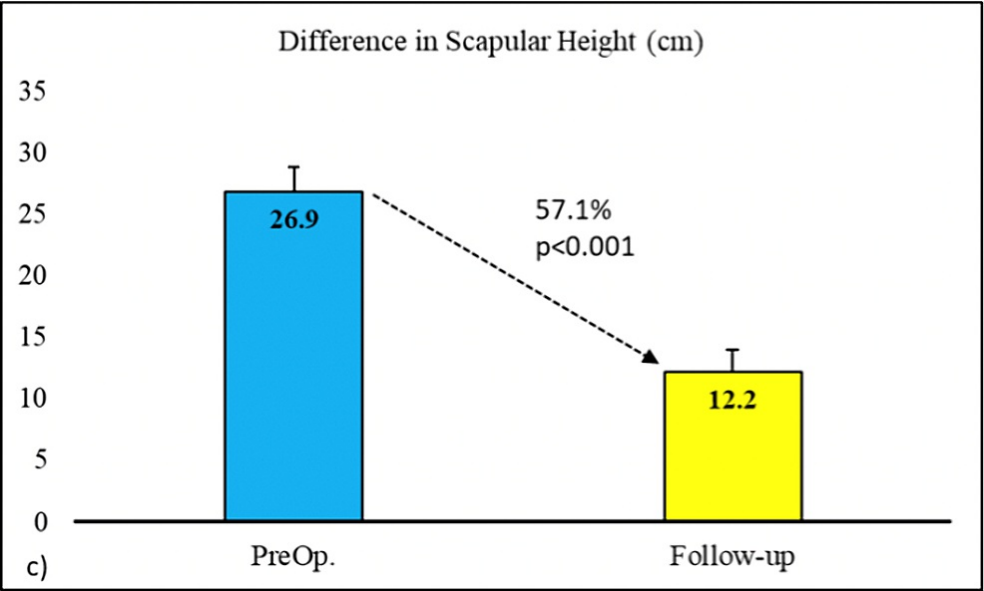
Difference in scapular heights (mm) at follow-up post-Woodward's procedure compared to preop state

The average preoperative Cavendish grade was 3.1 ± 0.6, which decreased to 1.3 ± 0.6 on the final follow-up with a percentage change of 58.3%. The decrease in Cavendish grade was found to be statistically significant (p<0.001). Furthermore, the average preoperative Rigault grade was 2.5 ± 0.5, which has decreased to an average of 1.8 ± 0.4 postoperatively with a percentage change of 24.1%. The change in Rigault grade was found to be statistically significant (p<0.001) (Figure 5). The average increase in the degree of shoulder abduction postoperatively was 48.3° with a percentage change of 53.8%. The increase in the arc of abduction was found to be statistically significant (p<0.001) (Figure 6). The average preoperative difference in scapular height (mm) was 26.9 ± 8.4 while it decreased to an average of 12.2 ± 7.5 on the final postoperative follow-up with an average change of 57.1%. The change in the difference in scapular height was found to be statistically significant (p<0.001) (Figure 7) (Table 2).

**Table 2:** Baseline characteristics of the study participants Note: Data are shown as N (%) for categorical variables (^); mean ± S.D. and range for normal continuous variables ($), and median (25th percentile, 75th percentile) and range for non-normal continuous variables (#).

	Total	
N	18 (100.0)	
Gender (^)		
Male	7 (38.9)	
Female	11 (61.1)	
Age Category (years) (^)		
2 to 3	5 (27.8)	
4 to 5	6 (33.3)	
6 to 7	4 (22.2)	
8 to 9	2 (11.1)	
24	1 (5.6)	
Side (^)		
Right	4 (22.2)	
Left	14 (77.8)	
Omovertebral Bar (^)		
+’ve	14 (77.8)	
-’ve	4 (22.2)	
	Average	Range
Age at Surgery (Years)#	4 (3,6)	2 - 24
Follow-up (Months) #	12 (9,18)	7 - 48
Preop. Cavendish Grade $	3.1 ± 0.6	2 - 4
Preop. Renault Grade $	2.5 ± 0.5	2 - 3
Preop. Shoulder Abduction $	93.3 ± 15.6	70 - 120
Preop. Difference in Scapular Height (mm) $	26.9 ± 8.4	15 - 44

**Figure 8:**
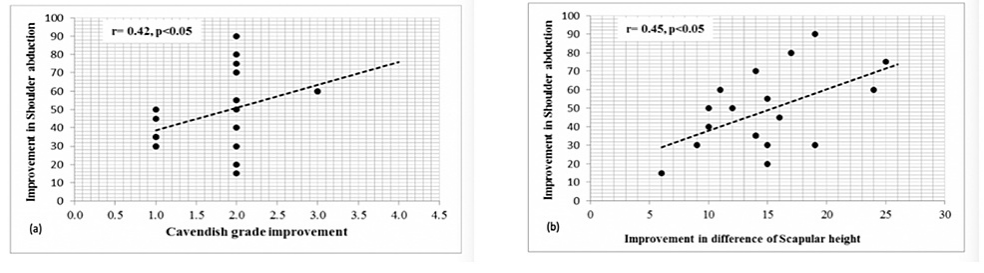
Scatter plots showing the correlation between a) Cavendish grade improvement and improvement in shoulder abduction, and b) improvement in shoulder abduction and improvement in difference of scapular height The dotted line shows the trend.

In addition, the study has shed highlight on observing the relationship between the different variables and their outcome. The study shows a positive correlation between improvement in the Cavendish grade and improvement in the shoulder abduction improves with both being positive (p<0.05, r=0.42). Moreover, the improvement in scapular height difference (mm) is also positively correlated with the improvement in the shoulder abduction (p<0.05, r=0.45) (Figure 8).

The adult outlier in our study who was a 24-year-old female had a preoperative Cavendish grade of 3 and a shoulder abduction of 95°, which changed to grade 2 and postoperative shoulder abduction of 130°, respectively.

In our study, 77.8% of the patients were found to have an omovertebral bar that was surgically excised during the surgical procedure. The Cavendish grade improvement in patients with omovertebral bar was 1.79 compared to 1.75 in patients without the bar (p=0.95). Rigault grade improvement was 0.71 in the positive group in comparison to 0.50 in the negative group (p=0.55). In regard to the improvement in the shoulder abduction, the patient's positive omovertebral group had an improvement of 47.86° in comparison to 50.0°in the negative group (p=0.81). However, the improvement in the difference of scapular height in the positive group was 14.43 mm compared to 15.75 mm in the negative group (p=0.65). Eventually, the study has concluded that the final outcome was not impacted by the absence or the existence of the omovertebral bar when measuring the outcomes in relation to the different variables (Table 3).

**Table 3:** Change at follow-up post-Woodward's procedure compared to preoperative Note: Data are shown as mean ± S.D. Change and % change at follow-up post-Woodward's procedure are indicated by either ↓ (reduced) or ↑ (increased). P-value is calculated by a paired sample t-test and indicates whether the change at follow-up is statistically significant (p<0.05).

Parameters	Preop.	Follow-up	Change	% Change	p
All Subjects (18)					
Cavendish Grade	3.1 ± 0.6	1.3 ± 0.6	↓1.8 ± 0.6	↓58.3 ± 18.1	<0.001
Renault Grade	2.5 ± 0.5	1.8 ± 0.4	↓0.7 ± 0.5	↓24.1 ± 21.6	<0.001
Shoulder Abduction	93.3 ± 15.6	141.7 ± 23.9	↑48.3 ± 21.1	↑53.8 ± 27.9	<0.001
Difference in Scapular Height (mm)	26.9 ± 8.4	12.2 ± 7.5	↓14.7 ± 4.9	↓57.1 ± 17.9	<0.001

**Figure 9:**
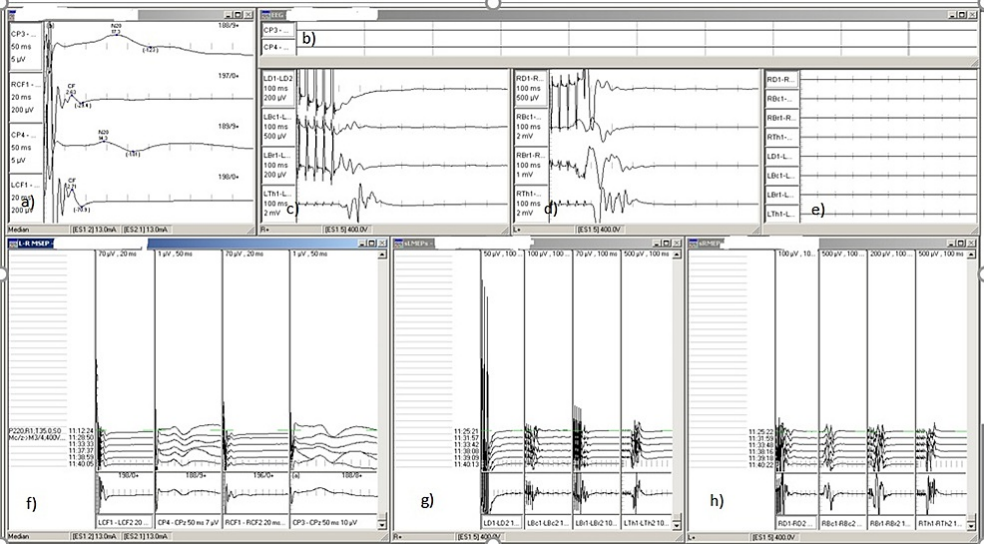
Intraoperative neuromonitoring in surgical correction of Sprengel's deformity of the left scapula in a three-year-old female patient a) SSEP tracing. Stimulation of left and right median nerve and recording from the peripheral and cortical area. b) EEG recording of the same patient. c&d) MEP recording from deltoid, biceps, brachioradialis, and thenar muscles of the left and right, respectively. e) Free-running EMG of the same patient. f) SSEP stack window. g&h) MEP left and right stack windows, respectively. SSEP: somatosensory evoked potential; EEG: electroencephalogram; MEP: motor-evoked potential; EMG: electromyogram

No significant changes during the recording of SSEPs and MEPs of brachial plexus neuromonitoring were noticed during the descent of the scapula (Figure 9).

Surgical correction of Sprengel's deformity has many potential complications. In the present study, eight (44%) patients had one or the other minor complications. Four patients had limitations of shoulder abduction. Delayed wound healing with superficial infection was seen in two patients. A hypertrophic scar was seen in one patient, whereas shoulder subluxation was seen in one of our patients.

## Discussion

Different surgical techniques are available for the management of Sprengel deformity, including Woodward’s procedure, Green’s procedure, Shrock’s procedure, Mear’s procedure, and partial scapulectomy [9-14]. In our study, we used Woodward’s procedure and additional neurophysiological monitoring of the brachial plexus, which has gained popularity recently, to prevent the risk of iatrogenic brachial plexus injury. The use of intraoperative neurophysiological monitoring during the variable surgical operations allows the neural integrity evaluation and provides a mapping of the nerves set at risk for iatrogenic injury during the procedure [2-3].

Ryabykh et al. in their study on 18 patients with Sprengel deformity used Woodward’s procedure and intraoperative neuromonitoring during the surgical procedure. The patients had good correction of the deformity, and no neurological deficit was noted postoperatively [2]. One of the aims of Woodward's procedure is to increase shoulder abduction. In the present study, we were able to achieve an average increase of 48.3° in shoulder abduction.

Elzohairy et al. [9] reviewed 10 patients who underwent Woodward’s procedure. The mean shoulder abduction improved from 83° to 152.5°, and the cosmetic appearance based on Cavendish grade has improved. Moreover, no complications have been observed except for one patient who had an unsightly scar. In our study, average shoulder abduction improved from 93.3° to 141.7°.

Crha and Gal [15], Carson et al. [16], Ross and Cruess [17], Grogan et al. [18], and Wu et al. [19], in their respective studies, noted significant improvement in shoulder abduction after Woodward's procedure.

Our results were similar to Carson et al. [16] who achieved an average increase of 50° in shoulder abduction. Walstra et al. [10] examined the data of seven patients treated with Woodward’s procedure; they have reported postoperative improvement of 56° in the shoulder abduction and a mean Cavendish grade improvement from grade 3 to 1-2.

In another study by Jindal and Gupta [8] reported the outcomes of the Woodward procedure in 12 patients. The shoulder abduction has improved to 153.3° with improvement in Cavendish grade of 1.25.

The authors also reported no difference in the final outcome related to the presence or absence of the omovertebral bar. The present study also concluded that there is no impact of the existence or nonexistence of omovertebral bar on the final outcome. Leibovich et al. [14] have noted the outcomes of modified Green’s procedure in a total of 18 patients. In a follow-up duration range of three years to 14 years and three months, cosmetic appearance has significantly improved, and the change of abduction was noted to improve to an average of 148°. However, two of the patients have developed partial recurrence that required reoperation. Additionally, we have reviewed the outcomes of Woodward’s procedure in multiple studies to evaluate and compare the different outcomes (Table 4).

**Table 4:** Demographics and results of patients of Sprengel’s deformity treated by Woodward's procedure by different authors

S no.	Study Author	Average age at Surgery	Number of Cases	Increase in Abduction (^o^)	Cavendish Grade Improvement
1	Jindal N, Gupta P [8]	5.6	12	37.5	1.92
2	Woodward JW [13]	8	9	32	-
3	Crha P, Gal B [15]	7	18	46	0.67
4	Carson WG et al [16]	9	11	29	-
5	Ross DM, Cruess RL [17]	-	17	35	-
6	Grogan DP et al [18]	6.5	20	37	-
7	Wu SJ et al [19]	4.7	9	78	2.23
8	Present Study	4	18	48.3	1.8

When the disease enters adulthood, the recommended procedure is resection of an omovertebral bone, the superomedial portion of the scapula, or both [5,12]. Minoru et al. [20] reported two adult patients, 20 and 26 years old, treated with resection of the omovertebral bone and the medial supraspinous region of the scapula. In the first patient, no significant improvement in shoulder abduction was achieved, whereas the second patient had improved shoulder abduction. We performed a standard Woodward procedure on a 24-year-old patient and achieved 130° of shoulder abduction at the seven-month follow-up compared to 95° preoperatively without any significant complications.

Complications are common in surgical correction of Sprengel's deformity, including hypertrophic scar, superficial and deep wound infections, iatrogenic brachial plexus injury, limitation of shoulder abduction, etc. [7]. Patwardhan et al. [11] in their study on 28 patients treated with Woodward’s procedure noticed only one case of delayed wound healing that healed subsequently with dressings and antibiotics. Elzohairy et al. [9] in their study of 10 patients reported one case of superficial infection and keloid formation. In the present study, we had two cases of delayed wound healing with a superficial infection, which healed subsequently with regular dressings and oral antibiotics. We also noticed a limitation of shoulder abduction in four of our patients, whereas one patient had a hypertrophic scar.

The limitations of our study were the small sample size and short follow-up duration. In addition, retrospective studies can have many types of biases, which could affect the results. In spite of the limitations mentioned, the results are promising and many meaningful inferences can be drawn from the study. Further studies taking into consideration these limitations will be very helpful.

## Conclusions

In our article, Woodward's procedure without clavicle osteotomy and using intraoperative neuromonitoring successfully corrects the deformity and improves the clinical outcome in the majority of patients with Sprengel's deformity even with a high-grade disease without any serious complications. Using intraoperative neuromonitoring in all cases of Woodward's procedure has been proven to be very helpful in assessing the brachial plexus and any abnormality detected during the procedure of scapular descent could be addressed intraoperatively.
